# Evaluation of Xpert MTB/RIF Ultra Assay for Diagnosis of Childhood Tuberculosis: a Multicenter Accuracy Study

**DOI:** 10.1128/JCM.00702-20

**Published:** 2020-08-24

**Authors:** Lin Sun, Yu Zhu, Min Fang, Yan Shi, Xiaoshan Peng, Qiong Liao, Xingyun Wang, Shuting Quan, Yacui Wang, Li Duan, Xiaomei Shi, Zhipeng Zhao, Lanqin Chen, Yongsheng Xu, Tongqiang Zhang, Xiaojian Cui, Chaomin Wan, Adong Shen

**Affiliations:** a Beijing Children’s Hospital, Capital Medical University, Beijing, China; b West China Second Hospital, Sichuan University, Chengdu, Sichuan, China; c No. 1 People’s Hospital of Liangshan Yizu Autonomous Prefecture, Liangshan, Sichuan, China; d Tianjin Children’s Hospital, Tianjin, China; UNC School of Medicine

**Keywords:** child, diagnosis, gastric aspirate, tuberculosis

## Abstract

A multicenter study was performed to evaluate the value of testing gastric aspirate (GA) with Xpert MTB/RIF Ultra assay (Ultra) for childhood tuberculosis (TB) detection in China. In total, 129 children with active TB and 173 children without TB were enrolled. The sensitivity of Ultra in bacteriologically confirmed TB and probable TB cases was 87.5% (42/48) and 44.4% (36/81), respectively. The specificity of Ultra was high (99.4%, 172/173). When Ultra, culture, and acid-fast bacilli outcomes were integrated as a composite reference standard, the percentage of children with definite TB increased from 37.2% (48/129) to 67.4% (87/129). The sensitivity of Ultra is 80.0% (40/50) in children aged <4 years, which is significantly higher than that in older children (48.1%, 38/79) (*P < *0.001). Ultra conducted using GA samples can provide faster results, allowing an early and accurate TB diagnosis, especially in younger children with difficulty producing sputum.

## Abstract (precis)

A multicenter study was performed to evaluate the value of testing gastric aspirate (GA) with Xpert MTB/RIF Ultra assay (Ultra) for childhood tuberculosis (TB) detection in China. In total, 129 children with active TB and 173 children without TB were enrolled. The sensitivity of Ultra in bacteriologically confirmed TB and probable TB cases was 87.5% (42/48) and 44.4% (36/81), respectively. The specificity of Ultra was high (99.4%, 172/173). When Ultra, culture, and acid-fast bacilli outcomes were integrated as a composite reference standard, the percentage of children with definite TB increased from 37.

## INTRODUCTION

The World Health Organization (WHO) estimated that there were 1.1 million new tuberculosis (TB) cases and 205,000 TB-related deaths among children in 2018 (1). There is a large gap between the global number of cases reported and the estimated incident cases, especially for high-TB-burden countries such as China. The gap is mainly due to the underdiagnosis and underreporting of TB cases. However, microbiological confirmation is often blocked by low bacterial loads, compounded by difficulties in obtaining appropriate samples from children.

Because of the limited value of microscopy and culture methods for TB detection in children, microbiological confirmation of childhood TB is rare, and clinical diagnosis depends mainly on contact history, clinical symptoms, and chest radiography (2). Culture confirmation can take several weeks due to such delayed microbiological results and the nonspecific clinical features of childhood TB; sometimes children who have underlying culture-confirmable pulmonary TB are misdiagnosed as having severe acute pneumonia and discharged without appropriate anti-TB treatment (3).

The WHO guidelines recommend the use of the Xpert MTB/RIF assay (Xpert) to improve TB and rifampin (RIF) resistance detection for childhood TB diagnosis (4). However, the sensitivity of this assay remains poor when the bacilli load is very low. To circumvent these limitations, a next-generation Xpert assay named the Xpert MTB/RIF Ultra assay (Ultra) has been developed. Ultra has been gradually introduced for TB diagnosis in adults, both in countries with a high TB prevalence (5, 6) and in those with a low TB prevalence (7, 8). However, data regarding its diagnostic accuracy in children are still lacking. Only a few reports have provided Ultra results that are relevant to the pediatric population, and all of the samples used in these studies were respiratory related, such as induced sputum (9–11), expectorated sputum (11), nasopharyngeal aspirates (10), and bronchoalveolar lavage fluid (12). Furthermore, no previous work has acquired samples via gastric lavage for Ultra as a means of detecting childhood TB.

For children who have difficulty producing sputum, alternate sample types used for Mycobacterium tuberculosis detection have included bronchoalveolar lavage fluid, gastric aspirate (GA), laryngeal swab, and stool samples (13, 14). GA samples appear to generate a higher detection rate than sputum specimens (acid-fast bacilli [AFB], 10.4% versus 5.7%; M. tuberculosis culture, 32.5% versus 17.9%) (15). Furthermore, GA samples show a superior diagnostic yield in non-sputum producers and in younger patients. However, there are limited data about the diagnostic value of GA samples in children with active TB. The present multicenter study was performed to retrospectively evaluate the value of performing Ultra using GA samples for detecting childhood TB in a high-TB-burden setting.

## MATERIALS AND METHODS

### Study population and samples.

This retrospective study was performed on samples obtained between 1 January 2018 and 30 November 2019 at the following three hospitals in China: Beijing Children’s Hospital (site A), West China Women’s and Children’s Hospital (site B), and the No. 1 People’s Hospital of Liangshan Yizu Autonomous Prefecture (site C). Children aged 15 years or younger were enrolled in the study if they had suspected symptoms of TB. A patient with suspected TB was defined as having TB based on the following factors: cough lasting for more than 2 weeks, weight loss, malnutrition, HIV, tuberculosis contact, and/or positive chest radiograph in accordance with the China and WHO guidelines.

The enrolled children were categorized into the following three groups (16): (i) bacteriologically confirmed TB—positive for culture of M. tuberculosis; (ii) probable TB—at least one TB symptom or sign and radiographic evidence consistent with TB and at least one of the remaining diagnosis criteria, including positive tuberculin skin test or interferon γ release assay result, clinical and radiological improvement seen following anti-TB chemotherapy, or documented exposure to TB; (iii) non-TB patients with respiratory tract infections (RTIs)—symptomatic but not fitting the above definitions and confirmed etiological evidence of infection with virus, mycoplasma, or bacteria.

This study was approved by the Ethics Committees of Beijing Children’s Hospital (number 2018-96). Written informed consent was obtained from the guardians of the patients.

### Procedures.

Briefly, gastric lavage was performed early in the morning after an overnight fast of at least 4 h. Based on age, 2 to 8 ml of GA was aspirated via a nasogastric tube. Each specimen was transported to the lab within 6 h of collection. GA was neutralized after transport to the lab. Acid-fast bacilli (AFB) microscopy was performed. The remaining GA specimens were stored at −80°C before being tested by Ultra (Cepheid, Sunnyvale, CA, USA) and M. tuberculosis culture. Mycobacterial culture was performed using mycobacteria growth indicator tubes (MGIT) and the Bactec 960 instrument (Becton, Dickinson, Sparks, MD, USA).

Preprocessing of the GA samples for use in Ultra was performed in accordance with the manufacturer’s instructions as previously described. GA samples were homogenized and digested using NaOH (final concentration, 1.5%) and vortexed at 5-min intervals for 15 min, with subsequent sample concentration at 4,000 × *g* for 15 min. The resulting supernatant was removed, and the remaining sediment was resuspended in 2 ml of phosphate buffer (pH 6.8) before being added to the sample chamber of the test cartridge. Test results evaluated the specimens as being invalid, not detected (negative), detected (positive with semiquantitation), or RIF resistant (detected, not detected, or indeterminate). The Ultra semiquantitative scale was set as trace, very low, low, medium, or high.

Demographic information and clinical data on the subjects were also collected, including their age, sex, history of contact with a TB patient, prior anti-TB treatment, and immunological test results.

### Statistical analysis.

The sensitivity and specificity of the assays were calculated using bacteriological results and clinical evidence as reference standards. To characterize the study population, we reported the numbers and percentages for categorical variables and the medians and interquartile ranges for continuous variables. McNemar’s test was used to evaluate differences in sensitivity and specificity; a *P* value of <0.05 was statistically significant. SPSS 23.0 software was used for statistical analysis.

## RESULTS

### Study participant characteristics.

**FIG 1:**
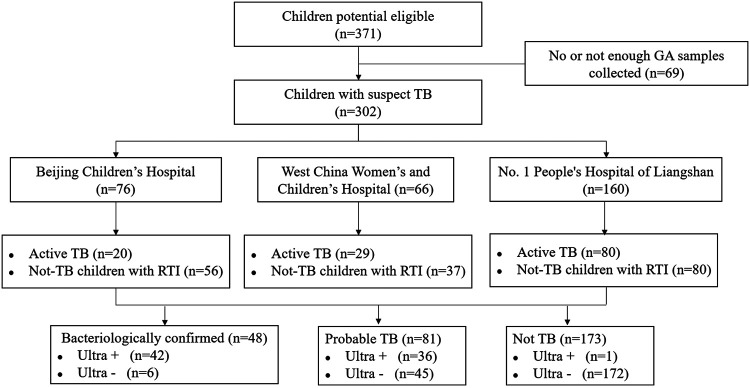
Flow chart of the study population.

In total, 371 children were initially enrolled at the three sites. Subsequently, 69 children were later excluded from the analysis due to having no or insufficient GA samples. Thus, the final case size used for our analysis was 302 children, which included 129 (42.7%) children with active TB (split between 48 bacteriologically confirmed cases and 81 probable cases) and 173 (57.3%) children without TB. The diagram in Fig. 1 shows the flow of participants according to the case definition categories and Ultra results.

Overall, 105 (34.8%) cases were in children under 4 years of age, and 215 (71.2%) cases were in children under 10 years of age. Furthermore, among children with active TB, 37.2% (48/129) of the children had culture-confirmed TB. Among the 81 children with probable TB, 5 (6.2%) had a positive AFB result. The demographic and clinical characteristics of all included cases are presented in Table 1.

**TABLE 1:** Main clinical characteristics of the study population

Characteristic	Total no. (%) (*n* = 302)	No. (%) bacteriologically confirmed TB (*n* = 48)	No. (%) probable TB (*n* = 81)	No. (%) non-TB (*n* = 173)
Mean age (interquartile range)	6.4 (2.1–10.7)	4.7 (0.8–9.7)	6.8 (2.8–10.7)	6.5 (2.1–10.6)
Gender				
Male	169 (56.0)	26 (54.2)	42 (51.9)	101 (58.4)
Female	133 (44.0)	22 (45.8)	39 (48.1)	72 (41.6)
Mycobacterium bovis BCG vaccination				
Yes	122 (40.4)	23 (47.9)	21 (25.9)	78 (45.1)
No	95 (31.5)	17 (35.4)	38 (46.9)	40 (23.1)
Unclear	85 (28.1)	8 (16.7)	22 (27.2)	55 (31.8)
Tuberculin skin test				
Positive	133 (44.0)	32 (66.7)	60 (74.1)	41 (23.7)
Negative	119 (39.4)	11 (22.9)	21 (25.9)	87 (50.3)
No data	50 (16.6)	5 (10.4)	0 (0)	45 (26.0)
Interferon γ release assay				
Positive	165 (54.6)	42 (87.5)	69 (85.2)	54 (31.2)
Negative	99 (32.8)	3 (6.3)	11 (13.6)	85 (49.1)
No data	38 (12.6)	3 (6.3)	1 (1.2)	34 (19.7)

### Pediatric TB diagnosis by Ultra.

Ultra testing was performed on GA samples from all 302 children. The resulting sensitivity was 60.5% (78/129) in children with active TB. Among the subset of 48 children with bacteriologically confirmed TB, Ultra testing yielded a higher sensitivity of 87.5% (42/48). Among the 81 children with probable TB, the sensitivity of the test was lower at 44.4% (36/81). Additionally, the specificity of Ultra was high (99.4%, 172/173).

**FIG 2:**
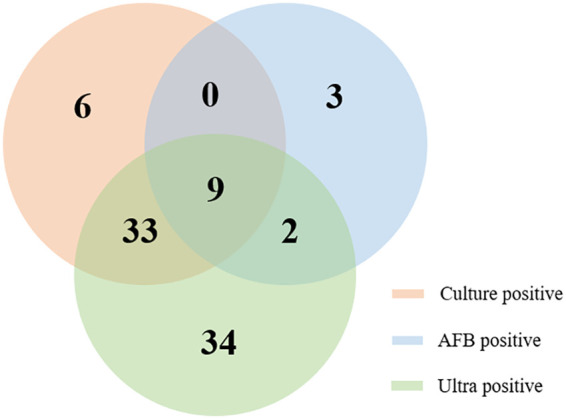
Venn diagram of the different diagnostic test results for childhood tuberculosis using gastric aspirate samples.

Among the 129 children with active TB, the sensitivity of bacterial culture and AFB was 37.2% (48/129) and 10.9% (14/129), respectively. In total, 87 children had a positive TB detection based on an assessment of their GA sample by any of the following three methods: culture, AFB, and/or Ultra (Fig. 2). When the Ultra, culture, and AFB outcomes were integrated as a composite reference standard, 39 of the 81 (48.1%) probable TB cases were reclassified as definite TB, and the percentage of children with definite TB increased from 37.2% (48/129) to 67.4% (87/129).

The sensitivities of Ultra and culture both varied between the three hospitals (Table 2). The percentage of children with culture-confirmed TB ranged from 27.5% (22/80) to 85.0% (17/20), and the sensitivity of Ultra ranged from 44.8% (13/29) to 80.0% (16/20). When the Ultra, culture, and AFB outcomes were integrated as a composite reference standard, the percentage of children with definite TB increased from 85.0% to 90.0% at site A, from 31.0% to 51.7% at site B, and from 27.5% to 67.5% at site C.

**TABLE 2:** Sensitivity of Xpert MTB/RIF Ultra in children with active tuberculosis enrolled in the multicenter study

Site and category	Total no.	No. positive	No. negative	Sensitivity (%)
Site A				
Bacteriologically confirmed TB	17	15	2	88.2
Probable TB	3	1	2	33.3
Total cases	20	16	4	80.0
Composite reference standard using culture, AFB, and Ultra	20	18	2	90.0
Site B				
Bacteriologically confirmed TB	9	7	2	77.8
Probable TB	20	6	14	30.0
Total cases	29	13	16	44.8
Composite reference standard using culture, AFB, and Ultra	29	15	14	51.7
Site C				
Bacteriologically confirmed TB	22	20	2	90.9
Probable TB	58	29	29	50.0
Total cases	80	49	31	61.3
Composite reference standard using culture, AFB, and Ultra	80	54	26	67.5

When the children with active TB were subgrouped according to age, the sensitivity of Ultra was 80.0% (40/50) in children younger than 4 years old, which is much higher than that in older children (48.1%, 38/79) (*P *< 0.001). The semiquantitative readout of Ultra (*P = *0.004) and the positive rate of M. tuberculosis culture (46.0% versus 31.6%; *P = *0.100) is higher in children younger than 4 years old. No difference was detected in the positive rate of AFB (10.0% versus 12.7%; *P = *0.646) by age.

Among the 78 children with positive Ultra results, the semiquantitative readout for positive Ultra samples was as follows: 5.1% high, 9.0% medium, 46.2% low, 14.1% very low, and 25.6% trace. The semiquantitative readout distribution was not significantly different between children with positive and negative culture results (*P = *0.211).

### RIF resistance detected by Ultra.

Among the 78 children with positive Ultra results, excluding the 20 children with “MTB detected, trace, indeterminate RIF resistance” results, 10 (17.2%, 10/58) specimens were detected to be RIF resistant. Among these 10 children, seven children had positive culture outcomes and phenotypic drug susceptibility testing results, including six RIF-resistant cases and one RIF-sensitive case.

## DISCUSSION

The laboratory diagnosis of childhood TB is hampered by several factors, including the difficulty in obtaining qualified samples and the low sensitivity of existing tests. Most children, especially young children, have difficulty producing sputum. In addition, children younger than 3 years of age are at high risk of rapidly progressing to severe forms of TB disease, such as miliary TB or TB meningitis (17). Therefore, the period for TB detection is shorter, and quick diagnostic tests are more urgent in children. Reevaluating and aggressively testing alternative nonrespiratory samples or using nonsputum-based methods could provide a more reliable diagnosis for childhood TB.

This study’s aim was to evaluate the accuracy of using Ultra on GA samples to diagnose TB in children. Here, GA sample sensitivity within the group of culture-confirmed TB cases was high (84.4%). When comparing the Ultra results with the reference standard of culture, the sensitivity with sputum samples ranged from 67.5% to 75% (9–11), that with nasopharyngeal aspirates was 52.5% (10), and that with bronchoalveolar lavage fluid samples was 91% (12). Although no data about the accuracy of Ultra on GA samples had been previously reported, studies using other molecular tests also suggested that GA samples have a superior diagnostic yield in non-sputum producers. A previous meta-analysis estimating the accuracy of Xpert for diagnosing pulmonary TB in children showed that the pooled sensitivity of GA samples was slightly higher than that of expectorated and induced sputum samples (66% versus 62%) (13).

The improved diagnostic effectiveness of Ultra in children was especially pronounced in culture-negative children; 46.5% of children with negative etiological results were detected as positive for TB by Ultra. Notably, when Ultra was integrated with culture and AFB results as a composite reference standard, the percentage of children classified as definite TB dramatically increased, especially for cases from site C, which had a lower percentage of culture-confirmed TB. We also found that the detection rate of Ultra and culture both varied between the three hospitals. To minimize the bias due to the operation of M. tuberculosis culture and molecular tests in this evaluation study, all of the samples from the three sites were tested at a central site. The samples from site A were tested within 3 days after collection, while samples from site B and C were frozen, transported, and tested within 2 months of collection. The variability of the sensitivity of both M. tuberculosis culture and Ultra may be attributed to the duration of storage of GA. One study reported that the detection rate of Xpert decreased with increasing duration of storage of GA (77.1% for 650 to 849 days and 50% for more than 1,050 days; *P *= 0.197) (18). However, contradictory observations about the effect of storage duration on the detection rate in sputum were reported in several studies. Tessema et al. reported that long-term storage had no significant effect on the rate of recovery of M. tuberculosis in all culturing systems (19). This is one of the limitations of our study. Further studies will be performed to analyze the effect of storage and transportation on the accuracy of Ultra.

Site C is a hospital from a remote mountainous area in western China with a high TB burden and limited resources. M. tuberculosis culture could not be performed on site there because the necessary equipment and facilities were not readily available. Consequently, all of the GA samples were transported to a specialized laboratory for M. tuberculosis culture. For sites like this one, a reliance on culture confirmation necessitates a lengthy delay in obtaining the etiological results. In contrast with these disadvantages of using culture, Ultra can be performed by a technician with minimal training at the same level of biosafety as microscopy. When the higher financial cost is not prohibitive, molecular tests are more useful for detecting childhood TB in cases with no or a negative etiological result.

The low proportion of TB detection in children aged less than 5 years is due not only to the paucibacillary nature of TB but also to the difficulties in obtaining a suitable respiratory sample (20). In this study, a higher sensitivity for Ultra is observed in children younger than 4 years than in older children, which suggests the particularly diagnostic value of Ultra in younger patients. Previous studies have reported that younger age and pulmonary and meningitic tuberculosis of children were associated with an increased yield for M. tuberculosis culture from gastric aspirates (21, 22). As the younger children cannot expectorate sputum, the bronchial secretions were usually swallowed, especially when sleeping, and the acid-resistant microorganisms were enriched in their GA. In addition, data from our hospital showed that younger children are at higher risk to progress to endobronchial tuberculosis and more likely to have multiple endobronchial lesions than older children (23). Interestingly, one 12-year-old child with suspected pleural TB yielded negative results when Ultra was performed on sputum, bronchoalveolar lavage fluid, and pleural fluid samples, whereas M. tuberculosis was finally detected by Ultra and culture when GA samples were used. These findings suggest that although GA samples are invasive to acquire, they are still an effective sample type for detecting M. tuberculosis in older children with highly probable TB.

The sensitivity of Ultra in this study is not perfect at only 59.5% across all TB patients. Due to the low bacteriological load in samples from children, multiple samples or multiple tests, including molecular or immunological tests, are needed to improve the diagnostic efficiency. Our study was designed to evaluate the testing of single specimens from children, which would save costs and reduce laboratory workload, so only one GA sample per patient was collected and tested. The sensitivity of Ultra found in this study is lower than that reported in our previous study, which utilized bronchoalveolar lavage fluid samples and yielded a sensitivity of 70% and 91%, respectively, in all pulmonary TB cases and in the bacteriologically confirmed TB cases (12). However, fiberoptic bronchoscopy to obtain bronchoalveolar lavage fluid samples must be performed by experienced physicians, and only children with indications should undergo fiberoptic bronchoscopy; in contrast, GA samples are easier to acquire in children.

Unlike studies that enrolled adult patients, our study on children had a higher proportion of Ultra results that were semiquantitatively evaluated as “trace.” This is consistent with the low M. tuberculosis load and bacteriological rates in children. Prior work found a positive correlation between Ultra semiquantitative results and smear microscopy results (24). Patients with high and medium positive Ultra results have a very high probability of being smear positive and have a high transmission potential, whereas those with low, very low, or trace positive Ultra results may have a relatively limited transmission potential. However, the potential correlation between Ultra results and transmission potential still needs further study, and the transmission potential of TB in children is important.

The dual abilities of Ultra testing to detect M. tuberculosis and to identify rifampin resistance is a further asset of this method. Multidrug-resistant TB is an increasing concern globally, not only in adults but also in children. Because the present work had a limited number of children whose samples were assessed by Ultra testing and also underwent drug susceptibility testing, no firm conclusion on the detection of RIF resistance can be reached from our data. However, other studies have reported that Ultra is a more efficient test for identifying cases with RIF resistance than other diagnostic methods (6, 25). To increase the sensitivity of RIF resistance detection, the Ultra incorporates melting temperature-based analysis. Four probes targeting RIF resistance mutations are also incorporated. If M. tuberculosis is detected with a “trace” result, then no interpretation can be made regarding RIF resistance, and results are reported as “MTB detected, trace, RIF indeterminate” because of the very low copies of the target gene. In our study, among the 78 children with positive Ultra results, 20 (25.6%) children had an “MTB detected, trace, indeterminate RIF resistance” result. This may be the limitation of Ultra in RIF detection.

In conclusion, this study demonstrated the relatively higher sensitivity of Ultra performed on GA samples for detecting M. tuberculosis in children. This method has diagnostic value for the early and accurate diagnosis of TB, especially in younger children who have difficulty producing sputum. Larger studies are required to determine the benefit of Ultra testing in a clinical setting.
